# Precision comparison of intensity ratios and area ratios in spectral analysis

**DOI:** 10.1038/s41598-024-71653-3

**Published:** 2024-10-15

**Authors:** Yuuki Hagiwara, Tatsu Kuwatani

**Affiliations:** Research Institute for Marine Geodynamics, Japan Agency for Marine-Earth Science and Technology, Yokosuka, 237-0061 Japan

**Keywords:** Cramér–Rao lower bound, Fisher information, Monte Carlo simulation, Intensity ratio, Area ratio, Variance–covariance matrix, Analytical chemistry, Geochemistry, Gas chromatography, Liquid chromatography

## Abstract

The long-debated question in analytical chemistry of which of the area ratio or the intensity ratio is the more precise has yielded no definitive analytical conclusion. To address this issue theoretically, we derived analytical solutions for the lower limits of estimation precision for spectral parameters, including the intensity ratio and area ratio, based on the Cramér–Rao lower bound (CRLB) framework for a Gaussian spectrum. The precisions of spectral parameter estimations from the analytical solutions were consistent with results obtained from Monte Carlo simulations. Our theoretical and simulation results revealed that the precision of estimating the area ratio surpassed that of the intensity ratio by a factor of $\sqrt{2}$. Additionally, our experimental results aligned well with both theoretical predictions and simulation outcomes, further validating our approach. This increased precision of the area ratio is due to negative covariance between intensity and bandwidth, rather than the area containing more intensity information, as often misinterpreted. Consequently, and quite counter intuitively, prior bandwidth and intensity related information does not improve the area ratio precision: it worsens it. The analytical solution we derived represents the fundamental limits of spectral parameter measurement precision. Thus, it can be used as an alternative method for estimating the minimum error when experimental measurement uncertainty cannot be determined.

## Introduction

In analytical chemistry, parameters extracted from spectra, such as intensity (*I*), peak position (*ω*_*c*_), full width at half maximum (FWHM) (*Γ*), area (*A*), wavenumber difference (Δ*ω*), intensity ratio (*R*_*I*_), area ratio (*R*_*A*_), and FWHM ratio (*R*_*Γ*_) are widely used as indicators of the physicochemical properties of substances. Among them, *R*_*I*_ and *R*_*A*_ (or *I* and *A*) have been used universally as explanatory variables in calibration curves because they are typical indicators of the concentrations of substances. Particularly, when fluctuations in intensity and area ratios are small, as in isotope labeling experiments^1–3^ and environmental monitoring using isotope ratios^4,5^, the analysis must be as precise as possible. In such situations, one crucial criterion for using either *R*_*I*_ or *R*_*A*_ (or *I* or *A*) as an explanatory variable is the precision of parameter estimation. Therefore, the question of which is the more precise has long been debated across various fields^6–14^.

However, experimental studies examining the precision of *R*_*I*_ and *R*_*A*_ (or *I* and *A*) have yielded inconsistent results. Some studies indicate that *R*_*I*_ (or *I*) is the more precise^15–17^, while others find *R*_*A*_ (or *A*) to be more precise^9,10^, with no definitive or quantitative conclusion reached. These discrepancies can be attributed to various factors, such as noise, spectral distortion, instrument performance, and uncertainties associated with population variance estimation, which vary from experiment to experiment. Although simulations and theoretical investigations that minimize these external factors are effective for providing a clear answer to the question, no such study has specifically contrasted the respective precisions of *R*_*I*_ and *R*_*A*_ (or *I* and *A*) using these methods.

To address this gap, our study employs a comprehensive approach that combines experimentation, simulation, and theoretical analysis to determine which of *R*_*I*_ or *R*_*A*_ (or *I* or *A*) offers better measurement precision. This novel approach, integrating theory and simulation, provides a quantitative answer to this long-standing question. Specifically, we derive analytical solutions for the estimation precision limits of spectral parameters based on the Cramér–Rao lower bound (CRLB) and validate these analytical solutions through Monte Carlo simulations. Subsequently, we compare the precisions of *R*_*I*_ and *R*_*A*_ (or *I* and *A*) obtained from the CRLB and simulations to determine which estimator is more precise. Additionally, we conduct experimental verification to evaluate whether the experimental data align with the theoretical and simulation results, thereby ensuring the robustness and practical applicability of our findings in real-world analytical contexts.

## Theory

Here we derive lower bounds on the precision of the estimation of the spectral properties of Gaussian functions that are characteristic of peaks strongly affected by Doppler broadening^18^ and instrument broadening^19^. Seven assumptions are made to derive an analytical solution for the lower bound of the estimation precision of the spectral characteristics (*θ* = (*I*,* ω*_*c*_, *Γ*)): (1) Noise variance equals the signal at all points; (2) The spacing (Δ*x*) of data points constituting the spectrum is constant over the frequency domain of interest; (3) The spectrum is nearly zero outside the sampling region; (4) The function is sampled closely enough in steps of Δ*x* that the sum is well represented by integration; (5) The baseline is constant; (6) The two peaks do not interfere with each other; (7) The noise at each data point is statistically independent and approximates a Gaussian probability density function (PDF) due to the sufficiently large average intensity. This approximation is valid according to the central limit theorem, which states that for large counts, Poisson-distributed noise can be effectively modeled by a Gaussian distribution. Several studies have explored the behavior of Gaussian parameter errors in the Gaussian noise limit^20–28^. Our derivation method is similar to that of Minin and Kamalabadi^22^, but with significant distinctions: (1) the noise is signal-dependent; (2) we establish an analytical solution for the error in the area; (3) we derive analytical solutions for the error in the ratio and difference of the estimated quantities; (4) we demonstrate how sample size affects precision. Hereafter, we use the term "error" to denote the deviation from known reference values, in accordance with metrological standards. When discussing measurement results where the true value is not known, we use the term "uncertainty" to describe the range within which the true value is expected to lie.

The Gaussian profile can be described as a function of intensity (*I*), peak center (*ω*_*c*_), and FWHM (*Γ*) as follows.

1 $$\gamma (\omega_{i}; \hat{\theta}) = I \exp \left[ \frac{- 4 \ln (2) \left( \omega_{i} - \omega_{c} \right)^2}{{\varGamma}^2} \right]$$

It is noteworthy that the unit of intensity is not a digital number (DN) (e.g., count), but an absolute physical unit (e.g., number of photons or electrons) that represents the characteristics of the Poisson noise. At high signal intensities, Poisson-distributed noise can be effectively approximated by a Gaussian distribution due to the central limit theorem. Because DN depends on the detector's analog-to-digital converter (ADC) sensitivity, using DN as a unit of intensity engenders incorrect estimator precision.

Let us consider a likelihood function pr(***γ***|*θ*), where the vector ***γ*** corresponds to the observed intensity profiles ***γ = ***[*γ*_1_, *γ*_2_, …, *γ*_*n*_] over *n* channels at ***ω = ***[*ω*_1_, *ω*_2_, …, *ω*_*n*_] and where *θ* = (*I*,* ω*_*c*_, *Γ*) denotes the parameter set we aim to estimate. Assuming that the distribution function in each channel is independent of every other distribution function, the likelihood (*L*) of a given parameter set producing a measured profile ***γ*** is

2 $$L = \text{pr} \left({\varvec\gamma} | \theta \right) = \mathop \prod \limits_{n = 1}^{N} \gamma(\omega_{n}; \hat{\theta}).$$

Taking the logarithm to obtain the log-likelihood,

3 $$l = \ln L = N \ln I - 4 \ln (2)\mathop \sum \limits_{n = 1}^{N} \left( \frac{\omega_{n} - \omega_{c}}{{\varGamma}} \right)^2.$$

The maximum likelihood estimate of the peak parameters is obtainable by maximizing Eq. (3).

The lower bound of the variance–covariance matrix (${\overline{\mathbb{C}}}$) is limited by the inverse of the Fisher information matrix (${\mathbb{F}}$) as

4 $${\overline{\mathbb{C}}} \ge {\mathbb{F}}^{ - 1} {,}$$

where Eq. 4 is known as the Cramér-Rao inequality^29,30^, meaning that there is a limit to the precision of experiments measuring the variable ${\hat{\theta }}$ via the emission model^31^. ${\mathbb{F}}$ is half of the Hessian matrix (${\mathbb{H}}$), with matrix elements given as^32^

5 $$F_{kl} = \frac{1}{2} H_{kl} = \frac{1}{2} \frac{\partial^2 \chi^2}{\partial \theta_{k} \partial \theta_{l}}\mathop \sum \limits_{{i}} \left( \frac{\partial \gamma(\omega_{i}; \theta)}{\partial \theta_{k}} \frac{\partial \gamma(\omega_{i}; \theta)}{\partial \theta_{l}} + \frac{\partial^2 \gamma(\omega_{i}; \theta)}{\partial \theta_{k} \partial \theta_{l}} R_{i} \right) \sigma_{i}^{-2} {,}$$

where *χ*^2^ is defined as the weighted sum of squares of the residuals (*R*_*i*_ ${{ = \gamma (\omega }}_{{i}} {; }\hat{\theta }{)} - {\gamma }_{{i}}$) between the intensity profiles ***γ*** detected in *n* channels corresponding to the wave number ***ω*** and Eq. (1). Because the second term in Eq. (5) can be ignored, the matrix elements of ${\mathbb{F}}$ can be approximated by $F_{kl} = \mathop \sum \limits_{{i}} \left( \frac{\partial \gamma(\omega_{i}; \theta)}{\partial \theta_{k}} \frac{\partial \gamma(\omega_{i}; \theta)}{\partial \theta_{l}} \right) \sigma_{i}^{-2}$^22,33,34^. Consequently, ${\mathbb{F}}$ for the ${\hat{\theta }}$ of the Gaussian function is expressed as presented below.

6 $$\mathbb{F} = \frac{1}{2} \sqrt{\frac{\uppi}{\ln 2}} \left( \begin{array}{ccc} \frac{\varGamma}{I \Delta x} & 0 & \frac{1}{\Delta x} \\ 0 & 8 \ln 2 \frac{I}{\varGamma {\Delta x}} & 0 \\ \frac{1}{\Delta x} & 0 & \frac{2I}{{\varGamma} \Delta x} \end{array} \right)$$

The corresponding variance–covariance matrix ${\overline{\mathbb{C}}}$ ($= {\mathbb{F}}^{ - 1}$) is

7 $$\overline{\mathbb{C}} = 2 \sqrt{\frac{\ln 2}{\uppi}} \left( \begin{array}{ccc} \frac{2 I \Delta x}{{\varGamma}} & 0 & - \Delta x \\ 0 & \frac{1}{8 \ln 2} \frac{{{{{\varGamma} \Delta x}}}}{{I}} &  0 \\ - \Delta x & 0 & \frac{{{{{\varGamma} \Delta x}}}}{{I}} \end{array} \right).$$

Note that Eq. (7) differs in both constant and variable terms from ${\overline{\mathbb{C}}}$ reported in the previous study^23^. One of the reasons is that they used Δ*x* ${{\gamma (\omega }}_{{i}} {; }\hat{\theta }{)}$ as *σ*_*i*_^2^ while we used ${{\gamma (\omega }}_{{i}} {; }\hat{\theta }{)}$. The lower bounds on the standard deviation of ${\hat{\theta }}$ are given as ${\sigma_{k}} {{ = \overline{C}}}_{{{kk}}}$ and are expressed as shown below.

8 $${\sigma_{I}}{ = }\left\{ {{4}\sqrt {\frac{{{\text{ln2}}}}{{\uppi }}} \frac{{{{I\Delta x}}}}{{\varGamma}}} \right\}^{{1/2}}$$

9 $${\sigma_{\omega_{c}}} { = }\left\{ {\frac{{1}}{{{4}\sqrt {{{\pi \text{ln2}}}} }}\frac{{{{\varGamma} {\Delta x}}}}{{I}}} \right\}^{{1/2}}$$

10 $${{\sigma_{\varGamma}}} { = }\left\{ {{2}\sqrt {\frac{{{\text{ln2}}}}{{\uppi }}} \frac{{{{{\varGamma} \Delta x}}}}{{I}}} \right\}^{{1/2}}$$

These results show that the variance of ${\hat{\theta }}$ is independent of the peak position and show that it improves with smaller Δ*x*. The relative standard deviations of *I*, *ω*_*c*_, and *Γ* are calculated from Eqs. (8–10).

11 $${\sigma^{*}_{I}} { = }\frac{{{\sigma_{I}} }}{I}{ = }\left\{ {{4}\sqrt {\frac{{{\text{ln2}}}}{{\uppi }}} \frac{{{\Delta x}}}{{{{{I\varGamma} }}}}} \right\}^{{1/2}}$$

12 $${\sigma^{*}_{\omega_{c}}} { = }\frac{{{\sigma_{\omega_{c}}}}}{{\omega_{c}} }{ = }\frac{{1}}{{\omega_{c}} }\left\{ {\frac{{1}}{{{4}\sqrt {{{\pi \text{ln2}}}} }}\frac{{{{{\varGamma} \Delta x}}}}{{I}}} \right\}^{{1/2}}$$

13 $${\sigma^{*}_{{\varGamma}}} { = }\frac{{{\sigma_{{\varGamma}}} }}{{{\varGamma}}}{ = }\left\{ {{2}\sqrt {\frac{{{\text{ln2}}}}{{\uppi }}} \frac{{{\Delta x}}}{{{{I{\varGamma} }}}}} \right\}^{{1/2}}$$

As intuition suggests, the relative standard deviation of the estimator ${\hat{\theta }}$ is proportional to (1/*I*)^1/2^. In addition, both ${\sigma^{*}_{I}}$ and ${\sigma^{*}_{{\varGamma}}}$ are proportional to {Δ*x/Γ*}^1/2^. In other words, the more data points exist above the FWHM, the better the precision obtained. However, ${\sigma^{*}_{\omega_{c}}}$ is proportional to {*Γ* × Δ*x*}^1/2^. Therefore, under the condition that Δ*x*/*I* = const., *I* and *Γ* can be estimated more precisely for wider peaks, whereas *ω*_c_ can be estimated more precisely for narrower peaks.

The intensity ratio (*R*_*I*_ = *I*_*w*_/*I*_*s*_), peak wavenumber difference (Δ*ω* =|*ω*_*w*_ − *ω*_*s*_|), and FWHM ratio (*R*_*Γ*_ = *Γ*_*w*_/*Γ*_*s*_) are often used as explanatory variables in calibration curves. These spectral parameters have consistently been utilized from early studies^35–37^ to recent research^38–40^ demonstrating their enduring importance in quantitative analysis across various fields. Here, the subscripts '*w*' and '*s*' respectively denote the spectral characteristics of the weaker and stronger peaks. Their standard deviations (${\sigma_{R_{I}}}$, *σ*_Δ*ω*_, and ${\sigma_{R_{{\varGamma}}}}$) are expressed respectively below using Eqs. (8–10) and standard error propagation.

14 $${\sigma_{R_{I}}} { = }\left\{ {{4}\sqrt {\frac{{{\text{ln2}}}}{{\uppi }}} \frac{{{I}_{{w}} }}{{{I}_{{s}}^{{2}} }}\left( {\frac{{{\Delta x}_{{w}} }}{{{{\varGamma}}_{{w}} }}{\text{ +} R}_{{I}} \frac{{{\Delta x}_{{s}} }}{{{{\varGamma}}_{{s}} }}} \right)} \right\}^{{1/2}}$$

15 $${\sigma_{\Delta \omega}} { = }\left\{ {\frac{{1}}{{{4}\sqrt {{{\pi \text{ln2}}}} {I}_{{w}} }}\left( {{{\varGamma}}_{{w}} {\Delta x}_{{w}} {\text{ +} R}_{{I}} {{\varGamma}}_{{s}} {\Delta x}_{{s}} } \right)} \right\}^{{1/2}}$$

16 $${\sigma_{R_{{\varGamma}}}} { = }\left\{ {{2}\sqrt {\frac{{{\text{ln2}}}}{{\uppi }}} \frac{{{{\varGamma}}_{{w}} }}{{{{\varGamma}}_{{s}}^{{2}} }}\left( {\frac{{{\Delta x}_{{w}} }}{{{I}_{{w}} }}{\text{ + }R}_{{{\varGamma}}} \frac{{{\Delta x}_{{s}} }}{{{I}_{{s}} }}} \right)} \right\}^{{1/2}}$$

From Eqs. (14–16), the relative standard deviations of ${\sigma_{R_{I}}}$, *σ*_Δ*ω*_, and ${\sigma_{R_{{\varGamma}}}}$ are expressed respectively as presented below.

17 $${\sigma^{*}_{R_{I}}}{ = }\frac{{{{\sigma_{R_{I}}} } }}{{{R}_{{I}} }}{ = }\left\{ {{4}\sqrt {\frac{{{\text{ln2}}}}{{\uppi }}} \frac{{1}}{{{I}_{{w}} }}\left( {\frac{{{\Delta x}_{{w}} }}{{{{{\varGamma}}_{{w}}} }}{\text{ +} R}_{{I}} \frac{{{\Delta x}_{{s}} }}{{{{\varGamma}}_{{s}} }}} \right)} \right\}^{{1/2}}$$

18 $${\sigma^{*}_{\Delta \omega}} { = }\frac{{{\upsigma }_{{{{\Delta \omega }}}} }}{{{{\Delta \omega }}}}{ = }\frac{{1}}{{{{\Delta \omega }}}}\left\{ {\frac{{1}}{{{4}\sqrt {{{\pi \text{ln2}}}} {I}_{{w}} }}\left( {{{\varGamma}}_{{w}} {\Delta x}_{{w}} {\text{ +} R}_{{I}} {{\varGamma}}_{{s}} {\Delta x}_{{s}} } \right)} \right\}^{{1/2}}$$

19 $${\sigma^{*}_{R_{{\varGamma}}}}{ = }\frac{{{{\sigma_{R_{{\varGamma}}}} } }}{{{R}_{{{\varGamma}}} }}{ = }\left\{ {{2}\sqrt {\frac{{{\text{ln2}}}}{{\uppi }}} \frac{{1}}{{{I}_{{w}} }}\left( {\frac{{{\Delta x}_{{w}} }}{{{{{\varGamma}}_{{w}}} }}{\text{ +} R}_{{I}} \frac{{{\Delta x}_{{s}} }}{{{{\varGamma}}_{{s}} }}} \right)} \right\}^{{1/2}}$$

According to Eqs. (17) and (19), when the second terms (i.e., $\frac{{{\Delta x}_{{w}} }}{{{{{\varGamma}}_{{w}}} }} \gg {R}_{{I}} \frac{{{\Delta x}_{{s}} }}{{{{\varGamma}}_{{s}} }}$) in parentheses are negligible, the primary parameters governing the relative standard deviations of *R*_*I*_ and *R*_*Γ*_ are the number of Δ*x* values that lie above the FWHM of the weak peak (*Γ*_*w*_/Δ*x*_*w*_) and the intensity of the weak peak (*I*_*w*_). It is important to note that, strictly speaking, the standard deviation is determined not merely by the number of data points above the FWHM, but by the number of Δ*x* values above the FWHM. Therefore, to improve the estimation precision of *R*_*I*_ and *R*_*Γ*_, the analytical conditions should be optimized to minimize Δ*x*_*w*_/*Γ*_*w*_*I*_*w*_. Because ${\sigma^{*}_{R_{I}}}$ and ${\sigma^{*}_{R_{{\varGamma}}}}$ are both represented by the same function except for the coefficient, their ratio is constant as

20 $$\frac{{{\sigma^{*}_{R_{I}}} }} {{{{\sigma^{*}_{R_{{\varGamma}}}} }} }{ = }\sqrt {2} .$$

Next, we derive a lower bound on the precision of estimating the area (*A*) under a spectrum. The area of the Gaussian function is a function of *I* and *Γ*, expressed as shown below.

21 $$A = \int_{-\infty}^{\infty} {I\text{exp}}\left[ {\frac{{ - {\text{4ln}}\left( {2} \right)\left( {{\omega} - {\omega }_{{c}} } \right)^{{2}} }}{{{{\varGamma}}^{{2}} }}} \right]{{d\omega }} = \frac{{1}}{{2}}\sqrt {\frac{{\uppi }}{{{\text{ln2}}}}} {{{I\varGamma}}}$$

**Fig. 1:**
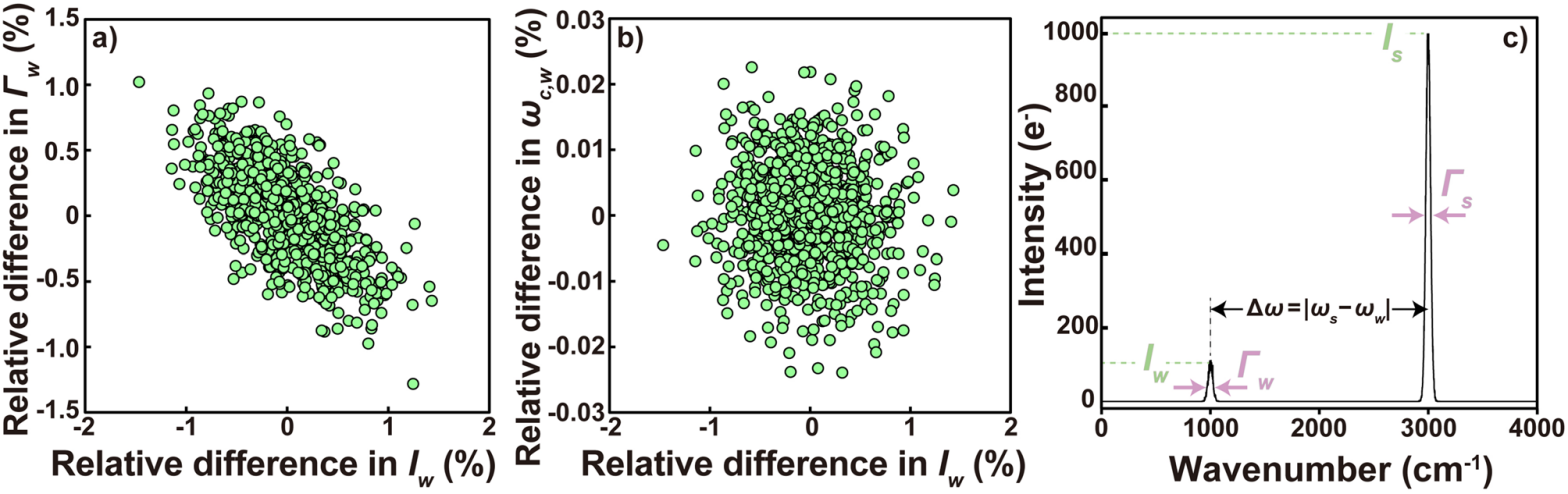
Correlations between (**a**) *I*_*w*_ and *Γ*_*w*_, and (**b**) between *I*_*w*_ and *ω*_*c, w*_ obtained from Monte Carlo simulations performed under the following conditions: Δ*x*_*s*_ = Δ*x*_*w*_ = 0.05 cm^−1^/pixel, *Γ*_*s*_ = *Γ*_*w*_ = 50 cm^−1^, *I*_*s*_ = 1000 e^−^, *I*_*w*_ = 100 e^−^, and Δ*ω* = 2000 cm^−1^. (**c**) Typical spectrum generated by the simulation.

Although the range of integration is assumed to be infinite in our theoretical calculations, note that in the actual analysis the integral range is finite and may affect the calculated peak area. From Eq. (21), Eq. (1) can be rewritten using $2 \sqrt{\frac{\ln 2}{\pi}} \frac{A}{{\varGamma}}$ instead of *I*. Because *A* is proportional to the product of *I* and *Γ*, the covariance between the two must be considered (Fig. 1a,b). The variance (${\sigma}_{{A}}^{{2}}$) of *A* is given as the ${\overline{\mathbb{C}}}$ of ${\hat{\theta }}$ and the gradient of *A* about ${\hat{\theta }}$^41^.

22 $${\text{var}}\left\{ {A} \right\}{{ = {\sigma}_{{A}}^{{2}} }} { = }\frac{{\partial {A}}}{{\partial {\hat{\theta }}^{{T}} }}{\overline{\mathbb{C}}}\frac{{\partial {A}}}{{\partial {\hat{\theta }}}}$$

Therein, $\frac{{\partial {A}}}{{\partial {\hat{\theta }}^{{T}} }}$ = ($\frac{{\partial {A}}}{{\partial {I}}}{,} \frac{{\partial {A}}}{{\partial {\omega }_{{c}} }}$, $\frac{{\partial {A}}}{{\partial {{\varGamma} }}}$)^*T*^. Therefore, the standard deviation (*σ*_*A*_) and relative standard deviation (σ*_A_) of *A* are expressed as

23 $${\sigma_{A}} { = }\left\{ {\frac{{1}}{{2}}\sqrt {\frac{{\uppi }}{{{\text{ln2}}}}} {{{I\varGamma} \Delta x}}} \right\}^{{1/2}} = \left\{ {{{A\Delta x}}} \right\}^{{1/2}} {\text{ and}}$$

24 $${\sigma^{*}_{A}} { = }\frac{{{{\sigma}}_{{A}} }}{{A}}{ } = \left\{ {\frac{{{\Delta x}}}{{A}}} \right\}^{{1/2}} .$$

Referring to Eqs. (11) and (24), we observe that the precision of *A* measurement is √2 times better than that of *I*. Using Eq. (23), the standard deviation of the area ratio (*R*_*A*_) is expressed by standard error propagation as presented below.

25 $${\sigma_{R_{A}}} = \left\{ {\frac{{{A}_{{w}} }}{{{A}_{{s}}^{{2}} }}\left( {{\Delta x}_{{w}} {\text{ +} R}_{{A}} {\Delta x}_{{s}} } \right)} \right\}^{{1/2}}$$

From Eq. (25), the relative standard deviation of ${\sigma_{R_{A}}}$ is

26 $${\sigma^{*}_{R_{A}}}{ = }\frac{{{{\sigma_{R_{A}} }} }}{{{R}_{{A}} }}{ = }\left\{ {{2}\sqrt {\frac{{{\text{ln2}}}}{{\uppi }}} \frac{{1}}{{{I}_{{w}} }}\left( {\frac{{{\Delta x}_{{w}} }}{{{{{\varGamma}}_{{w}}} }}{\text{ +} R}_{{I}} \frac{{{\Delta x}_{{s}} }}{{{{\varGamma}}_{{s}} }}} \right)} \right\}^{{1/2}} .$$

Therefore, because ${\sigma^{*}_{R_{A}}}$ is represented by the same function, except for the coefficients, as ${\sigma^{*}_{R_{I}}}$ and ${\sigma^{*}_{R_{{\varGamma}}}}$, the ratio of ${\sigma^{*}_{R_{I}}}$ to ${\sigma^{*}_{R_{A}}}$ is a constant as follows.

27 $$\frac{{{{\sigma^{*}_{R_{I}} }} }}{{{{\sigma^{*}_{R_{A}}}} }}{ = }\sqrt {2}$$

Equation (27) shows that *R*_*A*_ is a better estimator than *R*_*I*_, in terms of precision, for two Gaussian profiles that do not mutually interfere. This relation holds even when the values of* Γ* of the two peaks differ.

The relative standard deviation of all derived estimators is proportional to {Δ*x*/*I*}^1/2^ (Eqs. 11–13, 17–19, 24, and 26). For area detectors, binning a few pixels in both axes increases *I* at the expense of the sampling interval^42^. Performing *n* × *n* binning increases Δ*x* by a factor of *n*. The intensity of the spectrum after readout by full (or partial) vertical binning mode also increases by a factor of *n*. Therefore, {*n*Δ*x*/*nI*}^1/2^ = {Δ*x*/*I*}^1/2^, indicating that the precisions of the all estimators are neither improved nor deteriorated by binning at Poisson noise limit.

## Methods

### Monte Carlo simulation

We conducted Monte Carlo simulations to validate the analytical solutions. The simulation generated spectra with two Gaussian profiles separated by Δ*ω* = 2000 cm^−1^ (Fig. 1c). For this study, simulations were conducted under two sets of conditions: (1) Δ*x* = 0.05 cm^−1^/pix and *Γ* = 0.5, 1, 5, 10, and 50 cm^−1^ and (2) Δ*x* = 1 cm^−1^/pix and *Γ* = 2.5, 5, 10, and 50 cm^−1^, resulting in a total of nine conditions. Consequently, Δ*ω*/*Γ* is greater than 40. For the Gaussian function, intensity decreases by about 10^–11^ times at a distance of 3*Γ* from the peak center. Thus, the peak interference is negligible in our simulations. Under the simulation conditions, *Γ*/Δ*x* ranges from 2.5 to 1000. This implies that there are between 2.5 and 1000 Δ*x* above the FWHM. This condition applies to many analyses. The average intensities of the two peaks were fixed respectively as* I*_*s*_ = 1000 e^−^ and *I*_*w*_ = 100 e^−^. The variance of the total noise associated with each data point *γ* is given as *I*exp{− 4ln(2)(*ω*_*i*_ − *ω*_*c*_)^2^/*Γ*^2^}.

We generated 900 spectra for each parameter set. The extraction of spectral parameters was performed using a Python nonlinear fitting routine (scipy.optimize.curve_fit) that uses the Levenberg–Marquardt algorithm^43^. Known parameters served as initial guesses to facilitate a rapid convergence to the global minimum. All estimators obtained from a total of 8100 spectra are presented in Supplementary Table S1. Because our simulations have confirmed that all spectral characteristics are unbiased estimators, the CRLB enables us to estimate lower bounds on their variance (Supplemental Information and Supplementary Fig. S1).

To justify approximating the spectra generated by the simulations with Gaussian profiles, curve fitting was performed using both pure Gaussian and Pseudo-Voigt functions. The results showed that the parameter *x*, which represents the Lorentzian contribution, was practically zero, and the parameter estimates for intensity, peak position, width, and area were consistent between the two fitting methods, as detailed in Supplementary Table S2.

### Experimental validation

**Fig. 2:**
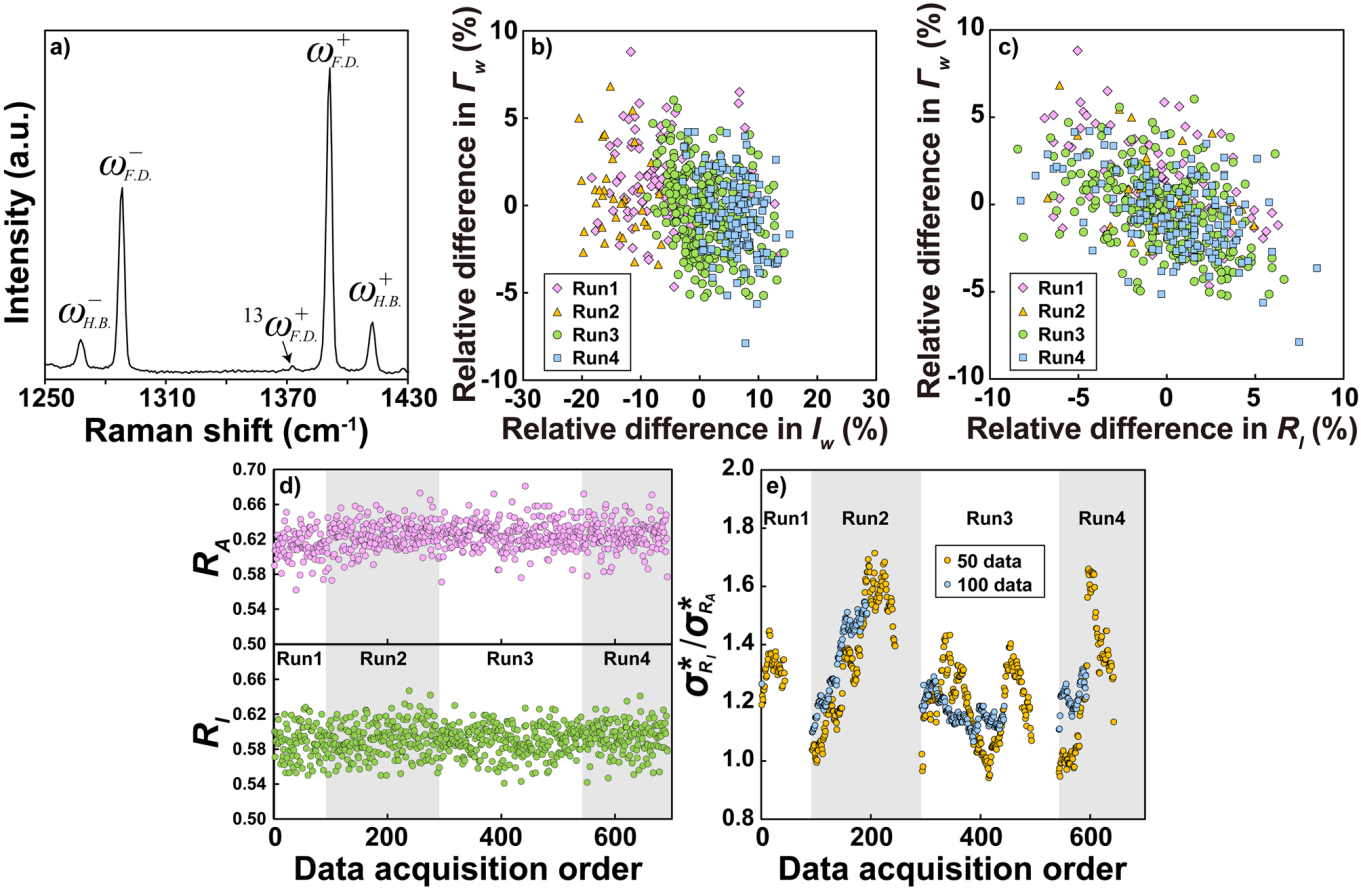
(**a**) Typical Raman spectrum of CO_2_ obtained from the experiment. Correlations between (**b**) *I*_*w*_ and *Γ*_*w*_ and (**c**) *R*_*I*_ and *Γ*_*w*_ across four experimental runs. (**d**) Plots of *R*_*A*_ and *R*_*I*_ obtained from repeated measurements over four sequential runs, displayed in the order of data acquisition. The shaded areas denote transitions between different runs. (**e**) ${\sigma^{*}_{R_{I}} } {{/{\sigma^{*}_{R_{A}} }}}$ plotted against the data acquisition order for four separate runs. Each point represents ${\sigma^{*}_{R_{I}} } {{/{\sigma^{*}_{R_{A}} }}}$. calculated using data from either 50 consecutive data (yellow circles) or 100 consecutive data (blue circles). Only '100 consecutive data' in Run 1 was calculated using 92 data points instead of 100, because only 92 data points were available.

To experimentally compare the precision of *R*_*A*_ and *R*_*I*_, we performed continuous Raman spectroscopic measurements of CO_2_ gas in a shrinkage bubble within a melt inclusion. This melt inclusion was sourced from an olivine sample of ocean island basalt collected off Rurutu, French Polynesia^44^. We selected CO_2_ gas for its minimal peak interactions and Gaussian-like peak shapes. As shown in Fig. 2a, the Raman spectrum of CO_2_ displays multiple peaks. Our primary analysis focused on the strong bands at approximately 1288 cm^−1^ (${\omega }_{F.D.}^{ - }$) and 1390 cm^−1^ (${\omega }_{F.D.}^{ + }$), known as the *v*_1_–2*v*_2_ Fermi diad^45^. The adjacent low-intensity peaks at approximately 1262 cm^−1^ (${\omega }_{H.B.}^{ - }$) and 1408 cm^−1^ (${\omega }_{H.B.}^{ + }$) are referred to as hot bands^46,47^. The peak at 1375 cm^−1^ (^13^${\omega }_{F.D.}^{ + }$) corresponds to the *v*_1_–2*v*_2_ Fermi resonance of ^13^C^16^O_2_^48^. We calculated the intensity and area ratios from the primary peaks of the Fermi diad, ${\omega }_{F.D.}^{ + }$ and ${\omega }_{F.D.}^{ - }$.

Measurements were performed using a micro-Raman spectrum analysis system (RAMANtouch VIS-HP-MAST; Nanophoton) at the Japan Agency for Marine-Earth Science and Technology. The sample was irradiated using a 532 nm diode-pumped solid-state laser through a 50 × objective lens (T Plan SLWD, Nikon Corp., N.A. = 0.8). The laser power was set at 29.3 mW and 37.4 mW, but no significant changes in *R*_*A*_ or *R*_*I*_ were observed due to differences in laser power. The backscattered Raman signal from the samples was channeled through a 100 μm diameter pinhole and slit to the spectrometer. The light entering the spectrometer was dispersed by a 1200 groove/mm diffraction grating. Photons reaching the detector were collected using a CCD camera with a pixel size of 20 μm (1340 × 400 pixels, PIXIS: 400B_eXcelon; Princeton Instruments). Data were collected at a sampling frequency of 100 kHz and output with a detector sensitivity of 1 e^−^/count. The spectra were collected in a single window ranging from 700 to 1901 cm^−1^. The pixel resolution (Δ*x*) was 0.90 cm^−1^/pixel near 1288 cm^−1^ and 0.89 cm^−1^/pixel near 1390 cm^−1^. Under the experimental conditions, *Γ*_*w*_/Δ*x*_*w*_ was approximately 3.6. During the analysis, room temperature, relative humidity, and indoor pressure were recorded using a data logger (TR-73H; T & D Corp.), with ranges of 21.7–22.2 °C, 43–51%, and 1001.8–1009.9 hPa, respectively (Supplementary Table S3).

Four consecutive measurements (Run 1–4) were conducted, with 92, 200, 250, and 150 analyses repeated for each run, respectively. The exposure time for each measurement was 15 s, and spectral data were acquired at approximately 20 s intervals (Supplementary Table S3). Data affected by spikes from cosmic rays were excluded, resulting in a total of 692 spectra used for precision evaluation. These data were processed using a Python nonlinear fitting routine, similar to the simulation, and the Pseudo-Voigt function was used for curve fitting. Supplementary Table S4 shows the results when the parameters *x*_*w*_ and *x*_*s*_, representing the contributions of the Gauss and Lorentz components, are treated as free parameters. Supplementary Table S3 shows the results when *x*_*w*_ and *x*_*s*_ are fixed at *x*_*w*_ = 0 and *x*_*s*_ = 0.29, the average values obtained in Supplementary Table S4. This suggests that the weaker peak, which predominantly influences the precision of the intensity and area ratios (Eqs. 17 and 26), can be approximated as a purely Gaussian profile given that *x*_*w*_ = 0, indicating no Lorentzian contribution. All spectral parameters obtained with both methods agreed within the error.

## Results

Our theoretical investigations, experiments, and simulations show that for well-separated peaks, the precision of *R*_*A*_ is superior to that of *R*_*I*_. Our simulation results and the derived variance–covariance matrix highlight the importance of the negative covariance between *I*_*w*_ and *Γ*_*w*_ in enhancing the precision of *R*_*A*_ over *R*_*I*_ (see Fig. 1a and the discussion below). If this negative covariance is also observed experimentally, it confirms its role in the higher precision of *R*_*A*_ over *R*_*I*_ in the experiment. Repeated measurements of the Raman spectrum of CO_2_ gas showed a weak negative correlation between *I*_*w*_ and *Γ*_*w*_, consistent with the simulation results (Fig. 2b). However, the absolute signal intensity varied greatly due to fluctuations in laser output and sample position (Supplementary Table S3). Therefore, the observed negative correlation between *I*_*w*_ and *Γ*_*w*_ might result from factors different from those in the simulations. In simulations without drift effects, data points consistently plotted in the same region of the *Γ*_*w*_–*I*_*w*_ space (Fig. 1a). If the experimental data were unaffected by drift, they would similarly plot in the same region across different runs. However, in practice, the plotted regions varied between runs (Fig. 2b), suggesting that the observed correlation between *I*_*w*_ and *Γ*_*w*_ in the experimental data might be an apparent trend caused by drift.

In contrast, a clear negative correlation between *Γ*_*w*_ and *R*_*I*_ was observed, with minimal variation between runs (Fig. 2c). Because the main source of variation in *R*_*I*_ is the high relative error of *I*_*w*_ rather than drift, the correlation between *R*_*I*_ and *Γ*_w_ effectively allows us to observe the underlying correlation between *I*_*w*_ and *Γ*_*w*_ without the confounding effects of drift. The clear negative correlation between *Γ*_*w*_ and *R*_*I*_ observed in both experimental results and simulations (Fig. 2c and Supplementary Fig. S2) supports this conclusion, reinforcing the idea that the precision of *R*_*A*_ is indeed higher than that of *R*_*I*_ due to the negative covariance between *I*_*w*_ and *Γ*_*w*_.

Despite the robustness of *R*_*A*_ and *R*_*I*_ against drift, slight changes were observed within the same run (Fig. 2d). To mitigate the impact of these fluctuations, we calculated the ratio ${\sigma^{*}_{R_{I}} } {{/{\sigma^{*}_{R_{A}} }}}$ for every 50 consecutive data points, as shown in Fig. 2e. This approach helped to minimize the effects of variability in *R*_*A*_ and *R*_*I*_. The results indicated that ${\sigma^{*}_{R_{I}} } {{/{\sigma^{*}_{R_{A}} }}}$ ranged from 0.94 to 1.71, with a mean of 1.27 and a standard deviation of 0.19 (Fig. 2e). These findings were consistent with both theoretical predictions and simulation results. Even when increasing the data segment size to 100 points, ${\sigma^{*}_{R_{I}} } {{/{\sigma^{*}_{R_{A}} }}}$ remained greater than unity (Fig. 2e). However, similar to previous studies, it remains challenging to draw strong quantitative conclusions about ${\sigma^{*}_{R_{I}} } {{/{\sigma^{*}_{R_{A}} }}}$ based on experimental data alone.

**Fig. 3:**
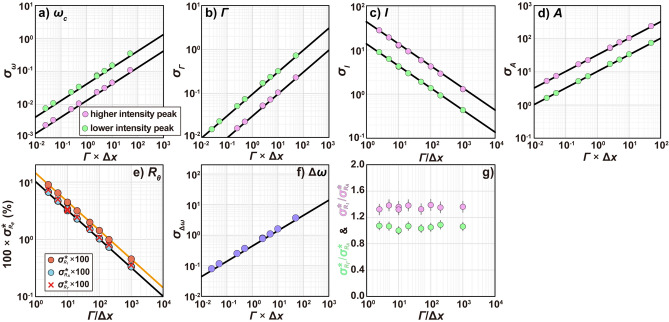
Standard deviation of (**a**) peak center, (**b**) FWHM, (**c**) intensity, (**d**) area, (**e**) relative intensity ratio, area ratio, and FWHM ratio, and (**f**) wavenumber difference. Solid black lines were computed using Eqs. (8–10, 15, 17, 19, 23, and 26). Symbols are the standard deviations (1*σ*) of estimators obtained from the simulations. (**g**) ${\sigma^{*}_{R_{I}} } {{/{\sigma^{*}_{R_{A}} }}}$ and ${\sigma^{*}_{R_{{\varGamma}}} } {{/{\sigma^{*}_{R_{A}} }}}$ obtained from the simulations. Error bars represent 95% confidence intervals.

Figure 3a–f show that ${\sigma_{\omega_{c}}}$,* σ*_*Γ*_,* σ*_*I*_,* σ*_*A*_, ${\sigma_{\Delta \omega}}$, ${\sigma^{*}_{R_{{\varGamma}}} }$, ${\sigma^{*}_{R_{I}} }$, and ${\sigma^{*}_{R_{A}} }$ obtained using the CRLB and simulations are in excellent agreement. This confirms the validity of Assumption (4), which states that the function is sampled closely enough in steps of Δ*x* that the sum is well represented by integration, under conditions where *Γ*/Δ*x* > 2.5. For highly discrete data with *Γ*/Δ*x* values considerably lower than 2.5, a different discrete mathematical approach might be more suitable. Additionally, the analytically obtained relation ${\sigma^{*}_{R_{I}} }:{\sigma^{*}_{R_{A}} }:{\sigma^{*}_{R_{{\varGamma}}} }$ = √2:1:1 is in line with the simulation results (Fig. 3g). To further evaluate the simulation results, we calculated 95% confidence intervals for the standard deviations of the spectral parameters obtained from the simulations and found that these intervals are smaller than the symbol size (Fig. 3a–f). However, the 95% confidence intervals for ${\sigma^{*}_{R_{I}} } {{/{\sigma^{*}_{R_{A}} }}}$ and ${\sigma^{*}_{R_{{\varGamma}}} }{{/{\sigma^{*}_{R_{A}} }}}$ are larger than the symbol sizes (Fig. 3g). Therefore, considering the large sample size required and the potential influence of disturbances such as drift, it is inferred that accurately determining ${\sigma^{*}_{R_{I}} } {{/{\sigma^{*}_{R_{A}} }}}$ and ${\sigma^{*}_{R_{{\varGamma}}} }{{/{\sigma^{*}_{R_{A}} }}}$ through experiments is not easy (Fig. 2e).

## Discussion

### Which is the more precise, area ratio or intensity ratio?

The debate on whether the intensity ratio or area ratio offers better precision for estimating objective variables has been a long-standing topic^6^ in analytical chemistry, frequently addressed in textbooks^8^ and review papers^7,11^. Previous studies suggest varying conclusions: the intensity ratio (or intensity) is sometimes more precise,^15–17,49^ while the area ratio (or area) is more precise in other instances.^9,10^ Some studies^50^ even find no significant difference between the two. Consequently, a definitive conclusion on this issue has not been established. Nevertheless, synthesizing the results of prior studies reveals a general trend: the intensity ratio (or intensity) offers better precision in cases of overlapping peaks or low signal-to-noise ratios, whereas the area ratio (or area) excels when peaks are distinctly separated.^7^ Therefore, our finding that the area ratio (or area) is √2 times more precise than the intensity ratio (or intensity) for well-separated Gaussian peaks is consistent with trends observed in previous studies.

Moreover, our previous study^17^, we demonstrated through a large-scale parameter study combining simulations and experiments that the intensity ratio is more precise than the area ratio when two peaks interfere with each other. Integrating these findings with our current results reveals that the superiority of the area ratio depends on the spectral characteristics of the target, particularly applying to well-separated peaks, but not universally to all conditions. This underscores the importance of considering the shape, interference, and noise of the peaks when deciding between intensity and area ratios for high-precision analysis.

The theoretical framework presented in this study can be extended to various conditions by appropriately substituting model functions (e.g., Lorentzian, Pseudo-Voigt, Voigt, Pearson VII) and noise conditions (e.g., Gaussian noise, flicker noise, blue noise) into Eq. (5) using ${{\gamma (\omega }}_{{i}} {; }\hat{\theta }{)}$ and *σ*_*i*_^2^. Furthermore, future research will aim to relax assumptions (1)–(7) (e.g., data discreteness and noise distribution) to develop a more broadly applicable theory under realistic and diverse conditions. The analytical solutions obtained through this approach can be used to estimate the minimum error in situations where it is challenging to experimentally assess uncertainty. Additionally, these analytical solutions can be utilized for evaluating the performance of developed data processing protocols and for providing guidance on where to invest in equipment components for high-precision analysis.

### Why area ratio precision is better: contributions of prior information and covariance

In specific cases, estimators can achieve greater precision when incorporating prior information^22,51,52^. Therefore, we conducted simulations to assess how the precision of estimating parameters changes when incorporating prior information. The spectra produced in the preceding section were fitted under the following three conditions: (1) optimizing all parameters, (2) optimizing while fixing *Γ* to a known value, and (3) optimizing while fixing *ω*_*c*_ to a known value.

The relative difference (100 × {${\sigma^{{\varGamma}\,fix}_{{\omega }_{{c}}}}$−${\sigma_{\omega_{c}}}$}/${\sigma_{\omega_{c}}}$)) in the error (${\sigma^{{\varGamma}\,fix}_{{\omega }_{{c}}}}$) of *ω*_c_ obtained when fixing *Γ* to a known value compared to the error (${\sigma_{\omega_{c}}}$) in *ω*_c_ when all variables are optimized, is − 0.3(7)%, showing no marked change (Supplementary Table S5). This is true because *ω*_*c*_ is independent of errors in other parameters^22^, as inferred from the fact that *F*_12_ = *F*_23_ = 0 in Eq. (6) and from Fig. 1b. Consequently, prior information about *I* and *Γ* does not enhance the precision of *ω*_*c*_ measurement. This point is also confirmed by the fact that the presence of a priori information related to *ω*_*c*_ does not change the errors in the estimates of the other parameters (Supplementary Table S5). Consequently, the CRLB of *ω*_*c*_ is simply obtained from the inverse of *F*_22_ (Eq. 6).

However, as can be inferred from Fig. 1a and ${\overline{C}}_{{{13}}}$ < 0 (Eq. 7), because the errors in *I* and *Γ* are not independent, *σ*_*I*_ decreases by 21.4(8)% when there is prior information in *Γ*. However, *σ*_*A*_ increases by 6.6(1.5)%. This phenomenon arises because *I* tends to decrease as *Γ* increases; also, *Γ* tends to decrease as *I* increases. Consequently, the product of *I* and *Γ* tends to be conserved at a constant value. In other words,* σ*_*A*_ is smaller than *σ*_*I*_ because of the negative covariance between *I* and *Γ*. As a result, when *Γ* is fixed at a known value, the relative difference of error (100 × {${\sigma^{* {\varGamma}\,fix}_{R_{I}}}$−${\sigma^{*}_{R_{I}} }$}/${\sigma^{*}_{R_{I}} }$) in *R*_*I*_ decreases by 21.1(6)%, whereas that in *R*_*A*_ increases by 6.6(1.8)%. Therefore, prior information related to *Γ* improves the precision of *R*_*I*_, but worsens the precision in *R*_*A*_. For that reason, prior information related to parameters should not be used if one wishes to estimate *R*_*A*_ as precisely as possible.

Our simulation results suggest the reason why previous and our experimental studies comparing ${\sigma^{*}_{R_{I}} }$ and ${\sigma^{*}_{R_{A}} }$ have had difficulty finding a quantitative relation between the two: First, determining an accurate ${\sigma^{*}_{R_{I}} } {{/{\sigma^{*}_{R_{A}} }}}$ requires a very large sample size. This is true because even our *n* = 900 simulations show some variation in $t{\sigma^{*}_{R_{I}} } {{/{\sigma^{*}_{R_{A}} }}}$ (Fig. 3g). Furthermore, conducting a large number of experiments without being affected by drift is challenging due to potential disturbances like fluctuations in room temperature and atmospheric pressure. Therefore, Monte Carlo simulations offer a significant advantage by allowing us to generate a large dataset under controlled conditions, thereby providing a robust validation of the theoretical predictions. Second, ${\sigma^{*}_{R_{I}} } {{/{\sigma^{*}_{R_{A}} }}}$ is not so different from unity.

### Variance of variance: why are small sample sizes likely to underestimate variance?

In practical analyses, precision is worse than the CRLB because of uncertainties arising from, errors in quantification coefficients^53^, peak-to-peak interference^54,55^, background noise^56^, and drift^17^, among other factors. Curiously, however, several earlier studies assessing the precision of *δ*^13^C measurements of CO_2_ by confocal Raman spectroscopy have reported analytical precision that is far superior to the limitations imposed by CRLB^17^. Because CRLB serves as a lower precision limit for estimators, this superior precision implies that the population variance was not accurately estimated in those earlier studies. Such results are expected to be attributable to over-optimized data processing (e.g., excluding results that are not inherently outliers from the analysis) or small sample sizes (*n*). Because the former cannot be evaluated based on information provided in earlier studies, we evaluate the latter in the following.

**Fig. 4:**
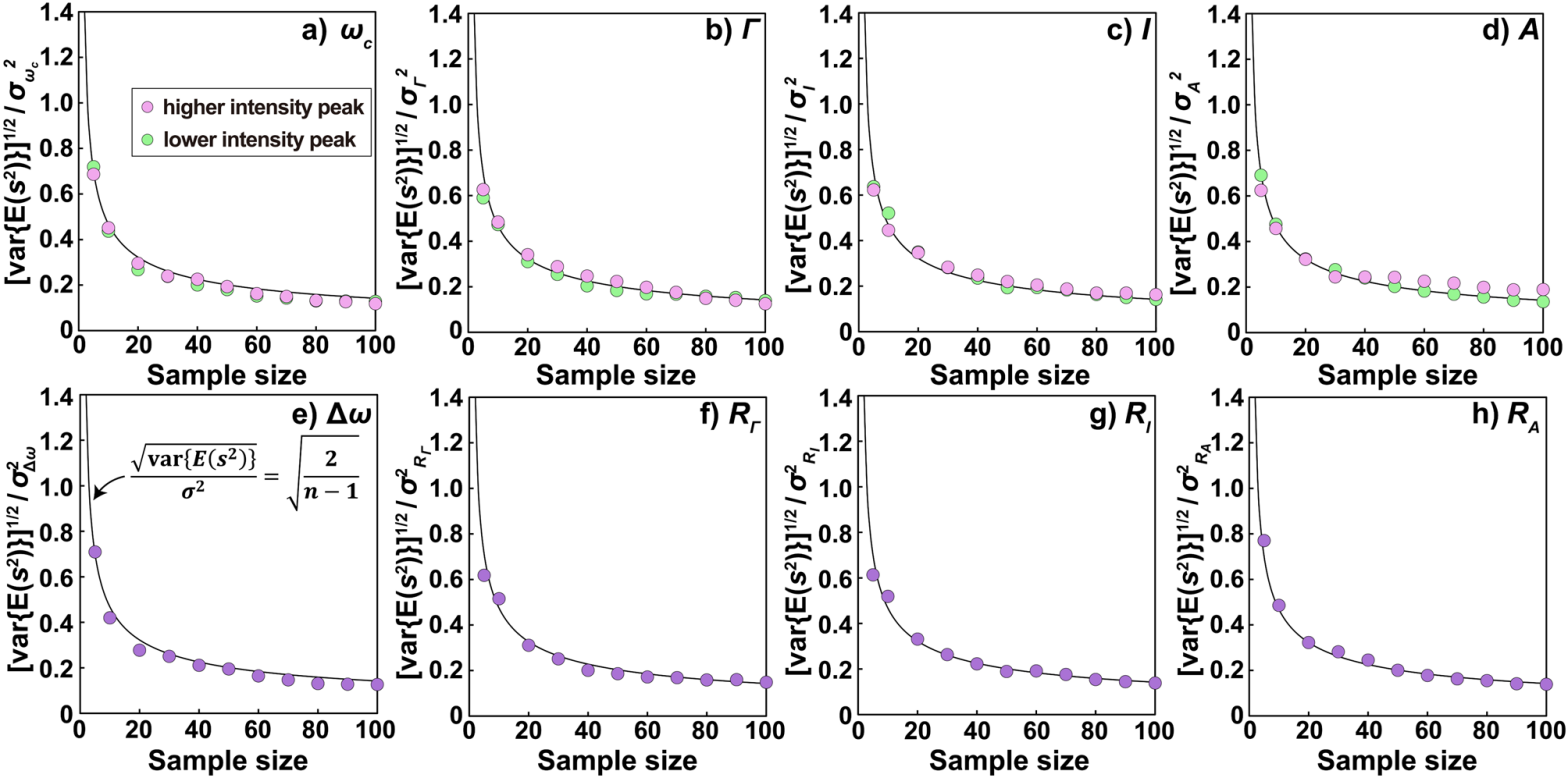
Relation between the square root of the variance of the expected value of unbiased variance (var[*E*(*s*^2^)]) divided by the population variance of the estimator (${{\sigma }}_{{{\hat{\theta }}}}^{{2}}$) and the sample size.

Through Monte Carlo simulation, we investigated the relation between the variance of the expected value of unbiased variance (var[*E*(*s*^2^)]) and sample size. The square root of var[*E*(*s*^2^)] divided by the population variance (${{\sigma }}_{{{\hat{\theta }}}}^{{2}}$) follows (2/*n* − 1)^1/2^ for all spectral characteristics (Fig. 4). This is true because, when *n* data points are drawn from the population to estimate the unbiased variance *s*^2^, var[*E*(*s*^2^)] follows the equation below^57^.

28 $${\text{var}}\left[ {{E}\left( {{s}^{{2}} } \right)} \right]{ = }\frac{{{{2{{\sigma }}_{{{\hat{\theta }}}}^{{4}}}}}}{{{n} - {1}}}$$

From Eq. (28), *s*^2^ becomes an unreliable estimator of ${{\sigma }}_{{{\hat{\theta }}}}^{{2}}$ when *n* is small. Furthermore, because *s*^2^ follows a chi-square distribution with *n* − 1 degrees of freedom, the mode of *s*^2^ is smaller than *E*(*s*^2^) when *n* is small (Supplementary Fig. S3). Therefore, whereas the *s*^2^ resulting from the experiments can be larger or smaller than ${{\sigma }}_{{{\hat{\theta }}}}^{{2}}$, the value of *s*^2^ estimated based on a small *n* tends to be smaller than ${{\sigma }}_{{{\hat{\theta }}}}^{{2}}$. This tendency may explain why earlier studies have documented measurement precisions of *δ*^13^C that are markedly smaller than the lower limit dictated by CRLB^17^. Therefore, when reporting experimental estimates of ${{\sigma }}_{{{\hat{\theta }}}}^{{2}}$, it is advisable to also report its confidence intervals (see supporting information and Supplementary Eq. S18 for details) using a *χ*-squared distribution with *n* − 1 degrees of freedom.

## Conclusions

To answer the question ‘Which is the more precise, intensity ratio (*R*_*I*_) or area ratio (*R*_*A*_)?’, we derived analytical solutions that establish the lower limit of precision for estimating the spectral characteristics (*I*, *ω*_*c*_, *Γ*, *A*, Δ*ω*, *R*_*I*_, *R*_*A*_, and *R*_*Γ*_) of the Gaussian profile under the Poisson noise limit based on the Fisher information and Cramér–Rao lower bound concepts. The results demonstrate that the precision of *R*_*A*_ is √2 times superior to that of *R*_*I*_. Similarly, the precision of *A* is √2 times better than that of *I*. The general trend based on the experimentally obtained results of earlier studies is that *R*_*A*_ and *A* are superior to *R*_*I*_ and *I* in terms of precision when the peaks are well separated. This observation was replicated in our experiments, with ${\sigma^{*}_{R_{I}} } {{/{\sigma^{*}_{R_{A}} }}}$ ranging from about 0.94 to 1.71. This study provides theoretical and quantitative evidence supporting this observation. Therefore, in terms of precision, *R*_*A*_ should be used rather than *R*_*I*_ when extracting physicochemical information from two well-separated Gaussian-shaped spectra. The challenge in providing a quantitative answer to this seemingly straightforward question through experimentation will be attributed to the fact that ${\sigma^{*}_{R_{I}} } {{/{\sigma^{*}_{R_{A}} }}}$ is close to 1.0. Moreover, an accurate estimation of ${\sigma^{*}_{R_{I}} } {{/{\sigma^{*}_{R_{A}} }}}$ necessitates the acquisition of a very large amount of spectral data under well-controlled conditions, making quantitative evaluation by experimentation difficult (Supplementary Table S1 and Eq. 28). When the peak shape, baseline model, noise condition, and Δ*ω*/*Γ* differ from those in this study, there is no guarantee that the results can be applied directly. However, in future work, our findings will be extrapolated to diverse conditions by substituting ${{\gamma (\omega }}_{{i}} ;{{\hat{\theta })}}$ and *σ*_*i*_^2^ appropriately following the analytical framework presented herein.

## Supplementary Information

Supplementary Information 1.

Supplementary Information 2.

## Data Availability

Data are provided within the manuscript or supplementary information files.
