# Supplementary material for: Precision comparison of intensity ratios and area ratios in spectral analysis
Source: Sci Rep. 2024 Oct 15;14:22898. doi: 10.1038/s41598-024-71653-3 (PMC11480322; doi:10.1038/s41598-024-71653-3)
Supplement: Supplementary file 2 — Supplementary Information 2. [file 41598_2024_71653_MOESM2_ESM.docx]

**Supplemental information**

**Precision comparison of intensity ratios and area ratios in spectral analysis**

Yuuki Hagiwara ^a *^ and Tatsu Kuwatani ^a^

^a^ Research Institute for Marine Geodynamics, Japan Agency for Marine-Earth Science and Technology, Yokosuka 237-0061, Japan

Email addresses and Orcid:

Email Orcid

Yuuki Hagiwara [hagiwaray@jamstec.go.jp](mailto:hagiwaray@jamstec.go.jp) 0000-0002-5515-0407

Tatsu Kuwatani [kuwatani@jamstec.go.jp](mailto:kuwatani@jamstec.go.jp) 0000-0001-7161-9458

* Corresponding author: [hagiwaray@jamstec.go.jp](mailto:hagiwaray@jamstec.go.jp) (Y. Hagiwara)

**Table of contents**

[S1. Theory 2](#_Toc173992035)

[S1.1 Details of the derivation of Fisher information matrix 2](#_Toc173992036)

[S1.2 Detailed calculation process from Eq. 7 to Eqs 17, 19, and 20 2](#_Toc173992037)

[S2. Monte Carlo Simulation 4](#_Toc173992038)

[S2.1 Bias of the estimators 4](#_Toc173992039)

[S2.2 Confidence interval 5](#_Toc173992040)

[S3. Supplementary discussion 5](#_Toc173992041)

[S3.1 Confidence intervals for experimentally estimated population variance 5](#_Toc173992042)

[Figure S1 7](#_Toc173992043)

[Figure S2 8](#_Toc173992044)

[Figure S3 9](#_Toc173992045)

# S1. Theory

## S1.1 Details of the derivation of Fisher information matrix

The derivatives of the Gaussian function profile with respect to the parameters *θ* = (*I*, *ω_c_*, *Γ*) are expressed as follows.

| $\frac{\text{∂}\gamma}{\text{∂}\text{I}}=\text{exp}\left( \frac{-\text{4}\ln\left( \text{2} \right)\left( \text{ω}-\text{ω}_{\text{c}} \right)^{\text{2}}}{\text{Γ}^{\text{2}}} \right)$ | (S1) |
| --- | --- |
| $\frac{\text{∂}\gamma}{\text{∂}\text{ω}_{\text{c}}}=\frac{\text{8}\ln\left( \text{2} \right)\text{I}\left( \text{ω}-\text{ω}_{\text{c}} \right)}{\text{Γ}^{\text{2}}}\text{exp}\left( \frac{-\text{4}\ln\left( \text{2} \right)\left( \text{ω}-\text{ω}_{\text{c}} \right)^{\text{2}}}{\text{Γ}^{\text{2}}} \right)$ | (S2) |
| $\frac{\text{∂}\gamma}{\text{∂}\text{Γ}}=\frac{\text{8}\ln\left( \text{2} \right)\text{I}\left( \text{ω}-\text{ω}_{\text{c}} \right)^{\text{2}}}{\text{Γ}^{\text{3}}}\text{exp}\left( \frac{-\text{4}\ln\left( \text{2} \right)\left( \text{ω}-\text{ω}_{\text{c}} \right)^{\text{2}}}{\text{Γ}^{\text{2}}} \right)$ | (S3) |

Equation S2 adds weights of equal magnitude but opposite signs to the Gaussian function at intervals [−∞, *ω_c_*] and [*ω_c_*, ∞], respectively. On the other hand, Eqs. S1 and S3 apply contrasting weights to the Gaussian function around *ω_c_*. Thus, the covariance between *ω_c_* and *I*, or *ω_c_* and *Γ*, converges to 0 by summing the first terms in Eq. 5 (i.e., *F*_12_ = *F*_23_ = 0). To approximate the sum of Eq. 5 by integration for computing each matrix element, we use the following equation.

| $\text{F}_{\text{ij}}\text{ ≈ }\frac{\text{1}}{\text{I}\text{Δ}\text{x}}\int_{-\infty}^{\infty} \frac{\text{∂}\text{γ}}{\text{∂}\theta_{\text{i}}}\frac{\text{∂}\text{γ}}{\text{∂}\theta_{\text{j}}}\frac{1}{\text{exp}\left( -\text{4}\ln\left( \text{2} \right)\left( \omega-\text{ω}_{\text{c}} \right)^{\text{2}}/\text{Γ}^{\text{2}} \right)}d\omega$ | (S4) |
| --- | --- |

By substituting Eqs. S1-S3 into Eq. S4, we obtain the following expressions for each matrix element.

| $\text{F}_{\text{11}}\text{ = }\sum_{\text{i}} \left( \frac{\text{∂}\text{γ}}{\text{∂}\text{I}} \right)^{2}\text{σ}_{\text{i}}^{\text{-2}}=\frac{1}{I}\sum_{\text{i}} \text{exp}\left( \frac{-\text{4}\ln\left( \text{2} \right)\left( \omega-\text{ω}_{\text{c}} \right)^{\text{2}}}{\text{Γ}^{\text{2}}} \right)=\frac{\text{1}}{\text{2}}\sqrt{\frac{\text{π}}{\text{ln2}}}\frac{\text{Γ}}{\text{I}\text{Δ}\text{x}}$ | (S5) |
| --- | --- |
| $\text{F}_{\text{22}}\text{ = }\sum_{\text{i}} \left( \frac{\text{∂}\text{γ}}{\text{∂}\text{ω}_{\text{c}}} \right)^{2}\text{σ}_{\text{i}}^{\text{-2}}=\frac{\text{64}\left( \text{ln2} \right)^{\text{2}}\text{I}}{\text{Γ}^{\text{4}}}\sum_{\text{i}} \left( \omega-\text{ω}_{\text{c}} \right)^{\text{2}}\text{exp}\left( \frac{-\text{4}\ln\left( \text{2} \right)\left( \omega-\text{ω}_{\text{c}} \right)^{\text{2}}}{\text{Γ}^{\text{2}}} \right)=\text{4}\sqrt{\text{πln2}}\frac{\text{I}}{\text{Γ}\text{Δ}\text{x}}$ | (S6) |
| $\text{F}_{\text{33}}\text{ = }\sum_{\text{i}} \left( \frac{\text{∂}\text{γ}}{\text{∂}\text{Γ}} \right)^{2}\text{σ}_{\text{i}}^{\text{-2}}=\frac{\text{64}\left( \text{ln2} \right)^{\text{2}}\text{I}}{\text{Γ}^{\text{6}}}\sum_{\text{i}} \left( \omega-\text{ω}_{\text{c}} \right)^{\text{4}}\text{exp}\left( \frac{-\text{4}\ln\left( \text{2} \right)\left( \omega-\text{ω}_{\text{c}} \right)^{\text{2}}}{\text{Γ}^{\text{2}}} \right)=\sqrt{\frac{\text{π}}{\text{ln2}}}\frac{\text{I}}{\text{Γ}\text{Δ}\text{x}}$ | (S7) |
| $\text{F}_{\text{12}}\text{ = }\sum_{\text{i}} \frac{\text{∂}\text{γ}}{\text{∂}\text{I}}\frac{\text{∂}\text{γ}}{\text{∂}\text{ω}_{\text{c}}}\text{σ}_{\text{i}}^{\text{-2}}=\frac{\text{8}\ln\left( \text{2} \right)}{\text{Γ}^{\text{2}}}\sum_{\text{i}} \left( \omega-\text{ω}_{\text{c}} \right)\text{exp}\left( \frac{-\text{4}\ln\left( \text{2} \right)\left( \omega-\text{ω}_{\text{c}} \right)^{\text{2}}}{\text{Γ}^{\text{2}}} \right)=\text{0}$ | (S8) |
| $\text{F}_{\text{13}}\text{ = }\sum_{\text{i}} \frac{\text{∂}\text{γ}}{\text{∂}\text{I}}\frac{\text{∂}\text{γ}}{\text{∂}\text{Γ}}\text{σ}_{\text{i}}^{\text{-2}}=\frac{\text{8}\ln\left( \text{2} \right)}{\text{Γ}^{\text{3}}}\sum_{\text{i}} \left( \omega-\text{ω}_{\text{c}} \right)^{\text{2}}\text{exp}\left( \frac{-\text{4}\ln\left( \text{2} \right)\left( \omega-\text{ω}_{\text{c}} \right)^{\text{2}}}{\text{Γ}^{\text{2}}} \right)=\frac{\text{1}}{\text{2}}\sqrt{\frac{\text{π}}{\text{ln2}}}\frac{\text{1}}{\text{Δ}\text{x}}$ | (S9) |
| $\text{F}_{\text{23}}\text{ = }\sum_{\text{i}} \frac{\text{∂}\text{γ}}{\text{∂}\text{ω}_{\text{c}}}\frac{\text{∂}\text{γ}}{\text{∂}\text{Γ}}\text{σ}_{\text{i}}^{\text{-2}}=\frac{\text{64}\left( \text{ln2} \right)^{\text{2}}\text{I}}{\text{Γ}^{\text{5}}}\sum_{\text{i}} \left( \omega-\text{ω}_{\text{c}} \right)^{\text{3}}\text{exp}\left( \frac{-\text{4}\ln\left( \text{2} \right)\left( \omega-\text{ω}_{\text{c}} \right)^{\text{2}}}{\text{Γ}^{\text{2}}} \right)=\text{0}$ | (S10) |

Thus, the Fisher information matrix for the parameter vector $\hat{\theta}$ of the Gaussian function is expressed as Eq. 6.

## S1.2 Detailed calculation process from Eq. 7 to Eqs 17, 19, and 20

In this section, we describe in detail the calculation procedure from Eq. 7 to Eqs. 17, 19, and 20. First, to obtain Eq. 17 (i.e., relative standard deviation (${\text{σ}\text{*}}_{\text{R}_{\text{I}}}$) of intensity ratio (*R_I_* = *I_w_*/*I_s_*)), we derive Eq. 14 (i.e., standard deviation ($\text{σ}_{\text{R}_{\text{I}}}$) of intensity ratio). From Eq. 8, because $\text{σ}_{\text{I}}=\left\{ \text{4}\sqrt{\frac{\ln\text{2}}{\text{π}}}\frac{\text{I}\text{Δ}\text{x}}{\text{Γ}} \right\}^{\text{1/2}}$, $\text{σ}_{\text{R}_{\text{I}}}$ is expressed as follows.

| $\text{σ}_{\text{R}_{\text{I}}}\text{ = }\left\{ \left( \frac{\text{σ}_{\text{I}_{\text{w}}}}{\text{I}_{\text{s}}} \right)^{\text{2}}+\left( \frac{\text{I}_{\text{w}}\text{×}\text{σ}_{\text{I}_{\text{s}}}}{\text{I}_{\text{s}}^{\text{2}}} \right)^{\text{2}} \right\}^{\text{1/2}}$ $\text{= }\left\{ \frac{\text{4}\sqrt{\frac{\ln\text{2}}{\text{π}}}\frac{\text{I}_{\text{w}}\text{Δ}\text{x}_{\text{w}}}{\text{Γ}_{\text{w}}}}{\text{I}_{\text{s}}^{\text{2}}}+\frac{\text{I}_{\text{w}}^{\text{2}}\text{×}\text{4}\sqrt{\frac{\ln\text{2}}{\text{π}}}\frac{\text{I}_{\text{s}}\text{Δ}\text{x}_{\text{s}}}{\text{Γ}_{\text{s}}}}{\text{I}_{\text{s}}^{\text{4}}} \right\}^{\text{1/2}}$ $\text{= }\left\{ \text{4}\sqrt{\frac{\ln\text{2}}{\text{π}}}\left( \frac{\text{I}_{\text{w}}\text{Δ}\text{x}_{\text{w}}}{\text{Γ}_{\text{w}}\text{I}_{\text{s}}^{\text{2}}}+\frac{\text{I}_{\text{w}}^{\text{2}}\text{Δ}\text{x}_{\text{s}}}{\text{Γ}_{\text{s}}\text{I}_{\text{s}}^{\text{3}}} \right) \right\}^{\text{1/2}}$ $\text{= }\left\{ \text{4}\sqrt{\frac{\ln\text{2}}{\text{π}}}\left( \frac{\text{I}_{\text{w}}^{\text{2}}}{\text{I}_{\text{s}}^{\text{2}}} \right)\left( \frac{\text{1}}{\text{I}_{\text{w}}} \right)\left( \frac{\text{Δ}\text{x}_{\text{w}}}{\text{Γ}_{\text{w}}}+\frac{\text{I}_{\text{w}}}{\text{I}_{\text{s}}}\frac{\text{Δ}\text{x}_{\text{s}}}{\text{Γ}_{\text{s}}} \right) \right\}^{\text{1/2}}$ | (S11) |
| --- | --- |

Here, the subscripts '*w*' and '*s*' respectively denote the spectral characteristics of the weaker and stronger peaks. Dividing $\text{σ}_{\text{R}_{\text{I}}}$ by *R_I_* to convert Eq. S11 to a relative standard deviation yields the following equation.

| ${\text{σ}\text{*}}_{\text{R}_{\text{I}}}\text{ }\text{= }\frac{\text{σ}_{\text{R}_{\text{I}}}}{\text{R}_{\text{I}}}\text{ }\text{}$ $\text{=}\frac{\left\{ \text{4}\sqrt{\frac{\ln\text{2}}{\text{π}}}\left( \frac{\text{I}_{\text{w}}^{\text{2}}}{\text{I}_{\text{s}}^{\text{2}}} \right)\left( \frac{\text{1}}{\text{I}_{\text{w}}} \right)\left( \frac{\text{Δ}\text{x}_{\text{w}}}{\text{Γ}_{\text{w}}}+\frac{\text{I}_{\text{w}}}{\text{I}_{\text{s}}}\frac{\text{Δ}\text{x}_{\text{s}}}{\text{Γ}_{\text{s}}} \right) \right\}^{\text{1/2}}}{\frac{\text{I}_{\text{w}}}{\text{I}_{\text{s}}}}\text{}$ $\text{=}\left\{ \text{4}\sqrt{\frac{\ln\text{2}}{\text{π}}}\frac{\text{1}}{\text{I}_{\text{w}}}\left( \frac{\text{Δ}\text{x}_{\text{w}}}{\text{Γ}_{\text{w}}}+\text{R}_{\text{I}}\frac{\text{Δ}\text{x}_{\text{s}}}{\text{Γ}_{\text{s}}} \right) \right\}^{\text{1/2}}$ | (S12) |
| --- | --- |

Eq. S12 is equal to Eq. 17. Thus, it was shown that Eq. 17 can be derived from Eq. 7.

Next, to obtain Eq. 19 (i.e., relative standard deviation (${\text{σ}\text{*}}_{\text{R}_{\text{Γ}}}$) of FWHM ratio (*R_Γ_* = *Γ_w_*/*Γ_s_*)), we derive Eq. 16 (i.e., standard deviation ($\text{σ}_{\text{R}_{\text{Γ}}}$) of FWHM ratio). From Eq. 10, because $\text{σ}_{\text{Γ}}=\left\{ \text{2}\sqrt{\frac{\ln\text{2}}{\text{π}}}\frac{\text{Γ}\text{Δ}\text{x}}{\text{I}} \right\}^{\text{1/2}}$, $\text{σ}_{\text{R}_{\text{Γ}}}$ is expressed as follows.

| $\text{σ}_{\text{R}_{\text{Γ}}}\text{ = }\left\{ \left( \frac{\text{σ}_{\text{Γ}_{\text{w}}}}{\text{Γ}_{\text{s}}} \right)^{2}+\left( \frac{\text{Γ}_{\text{w}}\text{×}\text{σ}_{\text{Γ}_{\text{s}}}}{\text{Γ}_{\text{s}}^{\text{2}}} \right)^{2} \right\}^{\text{1/2}}$ $\text{= }\left\{ \frac{\text{2}\sqrt{\frac{\ln\text{2}}{\text{π}}}\frac{\text{Γ}_{\text{w}}\text{Δ}\text{x}_{\text{w}}}{\text{I}_{\text{w}}}}{\text{Γ}_{\text{s}}^{\text{2}}}+\frac{\text{Γ}_{\text{w}}^{\text{2}}\times\text{2}\sqrt{\frac{\ln\text{2}}{\text{π}}}\frac{\text{Γ}_{\text{s}}\text{Δ}\text{x}_{\text{s}}}{\text{I}_{\text{s}}}}{\text{Γ}_{\text{s}}^{\text{4}}} \right\}^{\text{1/2}}$ $\text{= }\left\{ \text{2}\sqrt{\frac{\ln\text{2}}{\text{π}}}\left( \frac{\text{Γ}_{\text{w}}\text{Δ}\text{x}_{\text{w}}}{\text{I}_{\text{w}}\text{Γ}_{\text{s}}^{\text{2}}}+\frac{\text{Γ}_{\text{w}}^{\text{2}}\text{Δ}\text{x}_{\text{s}}}{\text{I}_{\text{s}}\text{Γ}_{\text{s}}^{\text{3}}} \right) \right\}^{\text{1/2}}$ $\text{= }\left\{ \text{2}\sqrt{\frac{\ln\text{2}}{\text{π}}}\left( \frac{\text{Γ}_{\text{w}}^{\text{2}}}{\text{Γ}_{\text{s}}^{\text{2}}} \right)\left( \frac{\text{1}}{\text{I}_{\text{w}}} \right)\left( \frac{\text{Δ}\text{x}_{\text{w}}}{\text{Γ}_{\text{w}}}+\text{R}_{\text{I}}\frac{\text{Δ}\text{x}_{\text{s}}}{\text{Γ}_{\text{s}}} \right) \right\}^{\text{1/2}}$ | (S13) |
| --- | --- |

Dividing $\text{σ}_{\text{R}_{\text{Γ}}}$ by *R_Γ_* = *Γ_w_*/*Γ_s_* to convert Eq. S13 to a relative standard deviation yields the following equation.

| ${\text{σ}\text{*}}_{\text{R}_{\text{Γ}}}\text{ = }\frac{\text{σ}_{\text{R}_{\text{Γ}}}}{\text{R}_{\text{Γ}}}\text{ }\text{}$ $\text{=}\frac{\left\{ \text{2}\sqrt{\frac{\ln\text{2}}{\text{π}}}\left( \frac{\text{Γ}_{\text{w}}^{\text{2}}}{\text{Γ}_{\text{s}}^{\text{2}}} \right)\left( \frac{\text{1}}{\text{I}_{\text{w}}} \right)\left( \frac{\text{Δ}\text{x}_{\text{w}}}{\text{Γ}_{\text{w}}}+\text{R}_{\text{I}}\frac{\text{Δ}\text{x}_{\text{s}}}{\text{Γ}_{\text{s}}} \right) \right\}^{\text{1/2}}}{\text{Γ}_{\text{w}}/\text{Γ}_{\text{s}}}\text{}$ $\text{=}\left\{ \text{2}\sqrt{\frac{\ln\text{2}}{\text{π}}}\frac{\text{1}}{\text{I}_{\text{w}}}\left( \frac{\text{Δ}\text{x}_{\text{w}}}{\text{Γ}_{\text{w}}}+\text{R}_{\text{I}}\frac{\text{Δ}\text{x}_{\text{s}}}{\text{Γ}_{\text{s}}} \right) \right\}^{\text{1/2}}$ | (S14) |
| --- | --- |

Eq. S14 is equal to Eq. 19. Thus, it was shown that Eq. 19 can be derived from Eq. 7.

Here, taking the ratio of Eq. S12 to Eq. S14, we obtain the following equation.

| $\frac{\text{σ*}_{\text{R}_{\text{I}}}}{\text{σ*}_{\text{R}_{\text{Γ}}}}\text{ = }\frac{\left\{ \text{4}\sqrt{\frac{\ln\text{2}}{\text{π}}}\frac{\text{1}}{\text{I}_{\text{w}}}\left( \frac{\text{Δ}\text{x}_{\text{w}}}{\text{Γ}_{\text{w}}}\text{ + }\text{R}_{\text{I}}\frac{\text{Δ}\text{x}_{\text{s}}}{\text{Γ}_{\text{s}}} \right) \right\}^{\text{1/2}}}{\left\{ \text{2}\sqrt{\frac{\ln\text{2}}{\text{π}}}\frac{\text{1}}{\text{I}_{\text{w}}}\left( \frac{\text{Δ}\text{x}_{\text{w}}}{\text{Γ}_{\text{w}}}+\text{R}_{\text{I}}\frac{\text{Δ}\text{x}_{\text{s}}}{\text{Γ}_{\text{s}}} \right) \right\}^{\text{1/2}}}\text{ }\text{=}\text{ }\sqrt{\text{2}}$ | (S15) |
| --- | --- |

Eq. S15 is equal to Eq. 20.

# S2. Monte Carlo Simulation

## S2.1 Bias of the estimators

Cramér-Rao lower bound represents the lower bound on the variance of any unbiased estimator. To determine if the estimated $\hat{\theta}$ from Monte Carlo simulation is an unbiased estimator, Fig. S1 illustrates the relationship between the bias (*B*$\left( \hat{\theta} \right)$) and *Γ*Δ*x* (or *Γ*/Δ*x*). Here, *B*$\left( \hat{\theta} \right)$ is defined by the following equation.

| $B\left( \hat{\theta} \right)\equiv E\left( \hat{\theta} \right)-\theta$ | (S16) |
| --- | --- |

When *B*$\left( \hat{\theta} \right)$ equals 0, the estimators are unbiased, and the PDFs of the estimators are exactly centered on the true values *θ*. Because *B*$\left( \hat{\theta} \right)$ were on average less than 5% of the standard deviation, the bias contributes little to the mean square error (Fig. S1). Consequently, all estimators are unbiased, and the CRLB enables us to estimate a lower bound on the variance.

## S2.2 Confidence interval

We calculated 95% confidence intervals for ${\text{σ}\text{*}}_{\text{R}_{\text{I}}}$/${\text{σ}\text{*}}_{\text{R}_{\text{A}}}$ and ${\text{σ}\text{*}}_{\text{R}_{\text{Γ}}}$/${\text{σ}\text{*}}_{\text{R}_{\text{A}}}$ (Fig. 3g). The confidence interval for the ratio of population variances was calculated using the following formula:

| $\text{F}_{\text{1}-\frac{\text{0.05}}{\text{2}}}\left( \text{899,899} \right)\text{×}\frac{{\text{s}\text{*}}_{\text{R}_{\text{I}}}^{2}}{{\text{s}\text{*}}_{\text{R}_{\text{A}}}^{2}}\leq\frac{{\text{σ}\text{*}}_{\text{R}_{\text{I}}}^{2}}{{\text{σ}\text{*}}_{\text{R}_{\text{A}}}^{2}}\text{ ≤ }\text{F}_{\frac{\text{0.05}}{\text{2}}}\left( \text{899,899} \right)\text{×}\frac{{\text{s}\text{*}}_{\text{R}_{\text{I}}}^{2}}{{\text{s}\text{*}}_{\text{R}_{\text{A}}}^{2}},$ | (S17) |
| --- | --- |

where ${\text{s}\text{*}}_{\text{R}_{\text{I}}}^{2}$ and ${\text{s}\text{*}}_{\text{R}_{\text{A}}}^{2}$ are the unbiased variances of the intensity ratio and area ratio for a sample size of *n* = 900. $\text{F}_{\text{1}-\frac{\text{0.05}}{\text{2}}}\left( \text{899,899} \right)$ and $\text{F}_{\frac{\text{0.05}}{\text{2}}}\left( \text{899,899} \right)$ are the values of the F-distribution with lower and upper tail probabilities of 1−0.05/2 and 0.05/2, respectively.

# S3. Supplementary discussion

## S3.1 Confidence intervals for experimentally estimated population variance

The CRLB we have derived can be used to verify whether the precision of the analysis under certain analytical conditions is asymptotically approaching theoretical limits, or to benchmark the performance of data processing algorithms or analysis protocols. However, as discussed in the main text, experimentally estimated population variances based on small sample sizes are unreliable and may not provide a meaningful contrast with the CLRB. In fact, local nondestructive *δ*^13^C measurements of CO_2_ using Raman spectrometry have reported much better precision than the precision limits imposed by the CLRB. This may be due to the small sample size, which prevents an accurate estimation of the population variance. Therefore, when comparing experimentally estimated population variances (or standard deviations) with CRLB or other experimental results, it is advisable to report confidence intervals for the population variances to account for the effect of sample size.

When the population variance *σ*^2^ follows a normal distribution, we can estimate a 100 × (1 − *α*)% confidence interval for the population variance as

| $\frac{\left( \text{n}-\text{1} \right)\text{s}^{\text{2}}}{\text{χ}_{\text{n}-\text{1, }\text{α}\text{/2}}^{\text{2}}}\text{ }\text{≤ }\text{σ}^{\text{2}}\text{ ≤}\text{ }\frac{\left( \text{n}-\text{1} \right)\text{s}^{\text{2}}}{\text{χ}_{\text{n}-\text{1, 1}-\text{α}\text{/2}}^{\text{2}}} ,$ | (S18) |
| --- | --- |

where *s*^2^ represents the unbiased sample variance and $\text{χ}_{\text{n}-\text{1, }\text{α}\text{/2}}^{\text{2}}$ can be calculated from the *χ*^2^ distribution table. Using Eq. S18, it is possible to more accurately compare the precision of experimental estimates of spectral characteristics with the CLRB derived in this study.

# Figure S1


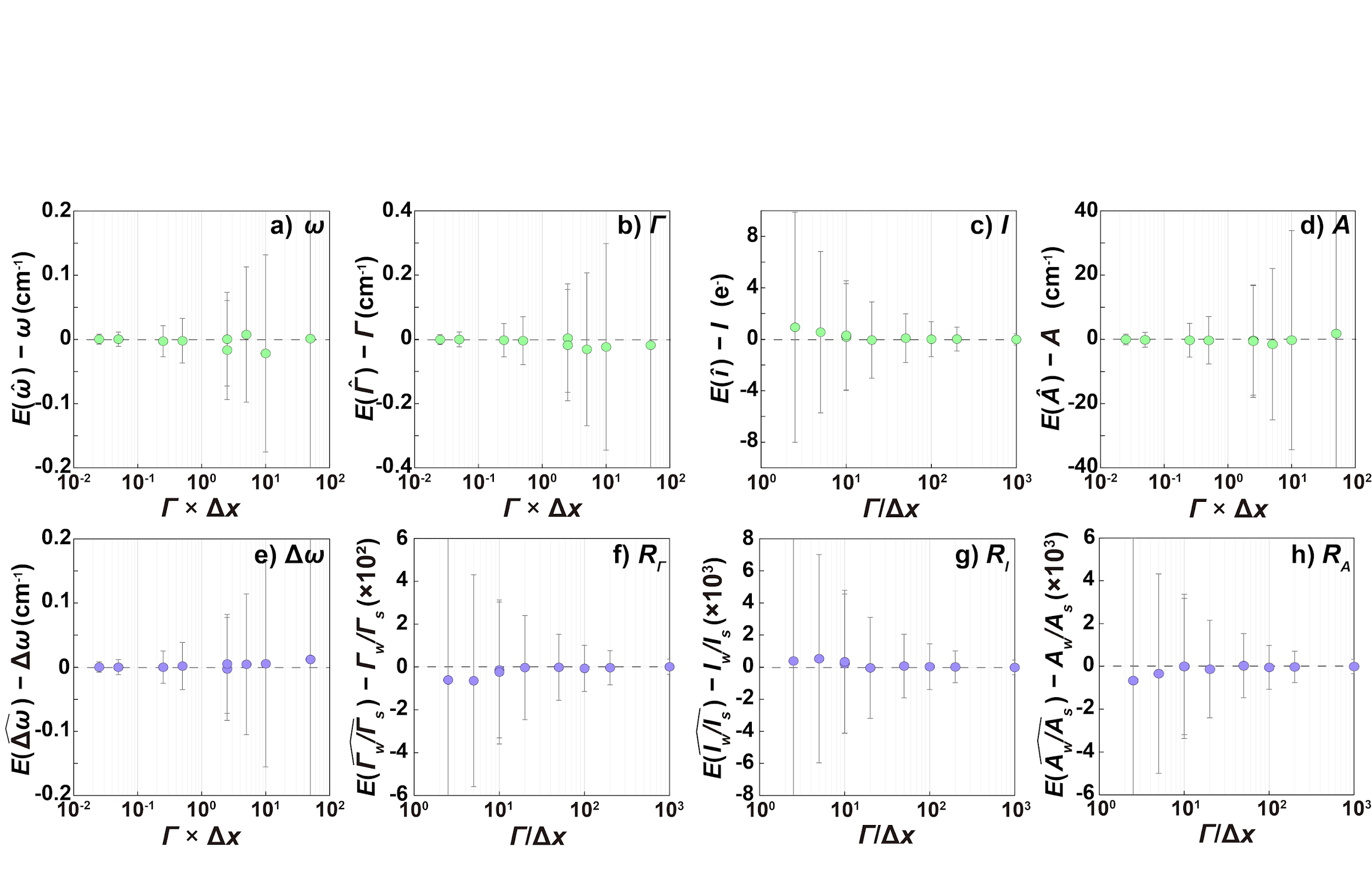
**Figure S1** Bias of the estimators. The vertical axis represents the difference between the expected value of the estimator ($E\left( \hat{\theta} \right)$) and the input value obtained from the simulation. For all simulation conditions, the bias of the estimator is well below its standard deviation.

# Figure S2


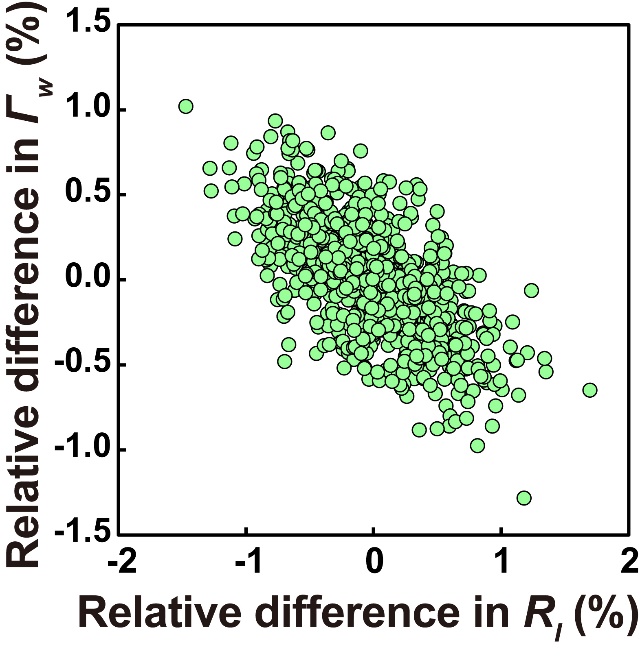


**Figure S2** Correlations between *R_I_* and *Γ_w_* obtained from Monte Carlo simulations performed under the following conditions: Δ*x_s_* = Δ*x_w_* = 0.05 cm^-1^/pixel, *Γ_s_* = *Γ_w_* = 50 cm^-1^, *I_s_* = 1000 e^-^, *I_w_* = 100 e^-^, and Δ*ω* = 2000 cm^-1^.

# Figure S3


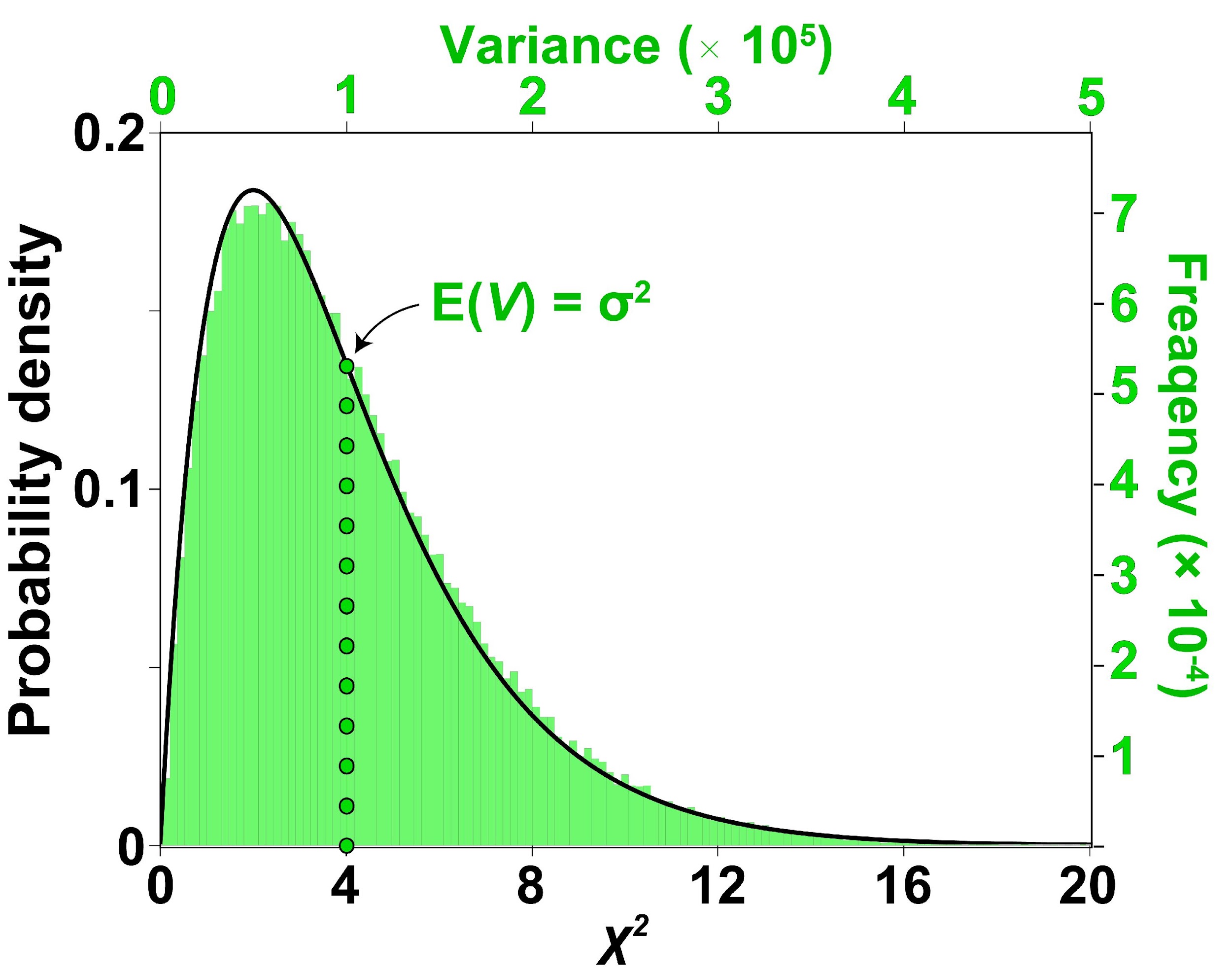


**Figure S3** The frequency distribution of the unbiased variance of intensity ratios, represented by green bins, is compared to the chi-square distribution (*n* = 5), depicted as a black solid line. The frequency distributions were obtained from 900 *R_I_* data sets generated by Monte Carlo simulations performed under the conditions *Γ* = 0.5 cm^-1^ and Δ*x* = 0.05 cm^-1^/pix. The expected value of the unbiased variance *E*[*Var*(*R_I_*)] ＝ 0.000102, and the unbiased variance $\sigma_{R_{I}}^{\text{2}}$ = 0.000103, as obtained from the Cramér-Rao lower bound of *R_I_* (Eq. 17), showing very good agreement (green dotted line).
